# Potential Utilization of Municipal Solid Waste Ash in Concrete Blends in Israel Part A: Municipal Waste Combustion in the Laboratory

**DOI:** 10.3390/ma19050969

**Published:** 2026-03-03

**Authors:** Sarit Nov, Shay Barak, Haim Cohen, Yaniv Knop

**Affiliations:** 1 Department of Chemical Sciences, Ariel University, Ariel 40700, Israel; 2 Department of Chemical Engineering, Ariel University, Ariel 40700, Israel; 3 Department of Chemistry, Ben Gurion University of the Negev, Beer Sheva 84105, Israel; 4 Department of Civil Engineering, Ariel University, Ariel 40700, Israel

**Keywords:** municipal solid waste ash (MSW ash), waste-to-energy, circular economy, incineration ash, concrete aggregates, waste valorization

## Abstract

This study investigated the potential use of ash derived from Municipal Solid Waste (MSW), typically destined for landfill in Israel, as a partial replacement for cement and aggregates in concrete mixtures, aligning with circular economy and sustainable construction objectives. MSW samples (post-metal and large plastic remains removal), supplied by the Dudaim Reclamation Center in Israel, were incinerated under controlled conditions in an upgraded laboratory furnace to produce ash. The ash content in the Israeli MSW was 18% ash. The ash consisted mainly of calcium-based minerals, including anhydrite (CaSO_4_), alite (3CaO·SiO_2_), and calcite (CaCO_3_), with minor quartz content, indicating potential pozzolanic behavior. The characterization results showed that appreciable amounts of ash produced from MSW incineration in Israel can be used as a partial replacement for cement and fine aggregates when properly treated. This study successfully established a laboratory-scale incineration process for Israeli MSW. The resulting ash was characterized, confirming its potential as a raw material for concrete applications, thereby paving the way for future studies on its performance as a partial substitute for cement and fine aggregates in concrete blends.

## 1. Introduction

The burgeoning global population and accelerated industrialization have led to an unprecedented increase in waste generation, posing significant environmental and resource management challenges worldwide. This escalating waste output, projected to increase by 70% globally by 2050 [1], necessitates innovative solutions beyond traditional disposal methods, which substantially contribute to greenhouse gas emissions [2]. One promising approach involves the valorization of waste materials, particularly municipal solid waste (MSW) ash, as a partial replacement for cement or fine aggregates in concrete mixtures, thereby aligning with circular economy principles and mitigating environmental impact [3,4]. A circular economy approach, which emphasizes the continuous use of products through recycling and reuse, is critical for sustainable development and reducing the negative environmental impacts associated with conventional construction practices [5]. By substituting Portland cement with MSW ash, this approach not only alleviates the environmental burden of cement manufacturing, such as significant CO_2_ emissions [6], but also addresses waste disposal challenges, creating a dual environmental benefit while conserving natural resources for power production using MSW and repurposing waste materials [7]. The increasing volume of waste generated owing to rapid population growth and technological advancements underscores the urgency of identifying sustainable waste management strategies [8]. The use of industrial by-products, such as MSW ash, as replacements for cement and fine aggregates in concrete production offers the dual benefit of minimizing waste-disposal challenges and reducing the environmental impact of cement manufacturing. In Israel, there is an increased problem of poor supply of fine aggregates [9], and if the MSW ash can also be used as a substitute, this would help in reducing the environmental damage accompanying the production of fine aggregates [10].

In parallel with the growing interest in MSW ash utilization, other major fractions of municipal solid waste have already been extensively investigated as secondary raw materials in cementitious systems. MSW is inherently heterogeneous and typically comprises organic matter, plastics, paper and cardboard, metals, textiles, and glass, with waste glass representing a significant and relatively stable inorganic fraction of urban waste streams. While color-sorted glass can be recycled, large quantities of mixed or contaminated waste glass are unsuitable for remelting and are therefore commonly landfilled or downcycled [11]. Consequently, finely crushed waste glass powder has attracted considerable attention as a construction material. Numerous recent studies [12,13,14] have examined urban waste glass as a reactive precursor or filler in alkali-activated materials (AAMs) and geopolymers, where its high amorphous silica content promotes dissolution under alkaline conditions and contributes to gel formation and strength development. In ordinary Portland cement (OPC) systems, ground waste glass has been widely studied as a supplementary cementitious material (SCM), while coarser fractions have been explored as fine aggregates [15]. When appropriate particle sizes and replacement levels are employed, waste glass can exhibit pozzolanic behavior and acceptable mechanical performance while mitigating alkali–silica reaction [16]. These studies establish crushed urban waste glass as one of the most mature and well-documented MSW-derived materials for OPC and AAM applications, thereby providing a useful benchmark for assessing less-explored MSW residues, such as municipal solid waste incineration ash.

Municipal solid waste incineration (MSWI) has become a well-established practice across the European Union, with 512 operational facilities reported in 2016 [17]. In conjunction with composting, recycling, and other waste management strategies, MSWI contributed to a reduction of nearly 60% (approximately 85 Mt) in the volume of municipal waste sent to landfills within the EU-28 countries, declining from 146 Mt in 1995 to 62 Mt in 2015 [17]. Over the same period, the total quantity of incinerated municipal solid waste in the EU-28 increased substantially, rising from 67 Mt to 137 Mt [18]. Currently, over 40% of MSW in the European Union, along with most of the incineration facilities, is utilized for energy recovery in the form of heat and electricity generation [19].

From a global perspective, it is crucial to examine specific national approaches. In Israel, for example, the predominant method for managing municipal waste, after the extraction of metals and plastics, is landfilling, with two large treatment and landfill sites located in the southern part of Israel in Dudaim and Efeh [20]. In Israel, the plan is to shift municipal solid waste treatment from reclamation to incineration as a better approach for MSW utilization.

The incineration of MSW produces two main types of solid residues: bottom ash and fly ash. The total ash produced is approximately 30% (by weight) of the incoming solid waste [21]. Typically, both ash types comprise CaO, SiO_2_, Al, Fe, Mg, Na, K, and Cl compounds, along with potentially toxic elements such as Cr, Ni, Cu, Zn, Cd, Hg, and Pb [21]. The final composition of the ash is largely determined by the nature of the waste mixture and the incineration process, which may vary by country. Given these diverse chemical and physical characteristics, MSW ash can be used as a suitable substitute for cement and aggregates in concrete. The municipal waste is not homogeneous, but the MSW ash produced via incineration is mixed, and thus the composition will be the same when large amounts of the MSW are incinerated.

Today, there is almost no practical implementation of ash generated from MSW incineration in the concrete industry, despite its demonstrated potential. Fly ash mainly contains lime, silicates, and alumina-silicates, which impart pozzolanic reactivity, enabling its use as a supplementary cementitious material in partial replacement of Portland cement [22]. Similarly, bottom ash has a granular texture and lower density than natural aggregates, allowing it to be processed into lightweight pellets or sintered products for use as coarse or fine aggregates. Experimental studies have shown that concrete incorporating treated MSW bottom ash can achieve compressive strengths of 20–25 MPa after 28 days [23]. In addition, the utilization of MSW ash in concrete offers significant environmental benefits by reducing landfill disposal, lowering raw material consumption, and promoting sustainable waste management practices [24]. However, there is still a lack of systematic laboratory-scale studies that correlate controlled incineration parameters with the chemical and physical properties of MSW ash relevant to concrete applications. Moreover, the composition of the ash is dependent on the nature of the municipal waste incinerated, and thus it is essential to study the typical municipal waste incineration in Israel. Two experimental incineration facilities (up to 300,000 tons of MSW per year) are planned to be built within the next 10 years. Laboratory studies are therefore essential to ensure a successful transition from reclamation to incineration in Israel, particularly to understand the unique characteristics of ash derived from local MSW incineration under controlled conditions. Accordingly, we developed and employed a reconstructed, improved laboratory incineration furnace to systematically investigate key combustion parameters and determine the quality of the resulting ash as a potential concrete component, thereby laying the groundwork for larger-scale assessments.

The results of the laboratory study are summarized in this publication.

## 2. Experimental Section

### 2.1. Materials

**Figure 1:**
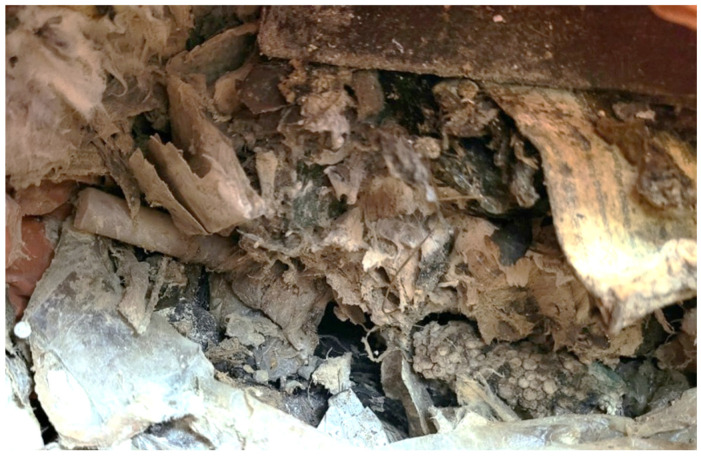
A sample of MSW intended for landfill at the Dudaim site.

The Dudaim Reclamation Center supplied municipal solid waste (MSW) for this study. (post separation of the metal waste and large plastic pieces), as shown in Figure 1.

### 2.2. Analysis and Methods

XRF analysis: The ash samples were analyzed using a Zetium XRF spectrometer by Panalytical Company (Malvern, UK) at the laboratory of Environmental Services Company Ltd. (Neot Hovav, Israel).

SEM analysis: The ash samples were analyzed using an Ultra-High Resolution Maia 3 FE-SEM microscope by Tescan Company (Brno, Czech Republic) at the Surface Laboratory of Ariel University. The microscope operates at an accelerating voltage of approximately 10 kV with a secondary electron detector.

XRD analysis: The ash samples were analyzed using an X’pert Pro X-ray diffractometer by PANalytical Company (Malvern, UK) at the Surface Laboratory of Ariel University. XRD patterns were collected using a Cu Kα radiation source (λ = 1.5406 Å) in θ–2θ geometry over a 2θ range of 5–70°, with a step size of 0.01° and a counting time of 1 s per step (scan rate 0.6° min^−1^). Measurements were performed at room temperature using fixed divergence and receiving slits.

As there is an essential problem of homogeneity of the municipal waste, the incineration process of the municipal waste has been checked in several samples (including the moisture content of the waste prior to the incineration), whereas the initial incineration process was in all experiments at the same 850 °C and the air flux into the quartz reactor was kept constant, and indeed the results had some variations (see Section 3), and thus we have averaged the resulting parameters (e.g., moisture content, ash content, etc.).

## 3. Results and Discussion

In order to incinerate MSW, the regular furnace available could not be used and had to undergo substantial changes. Also, the moisture content of the MSW interferes with the operation of the upgraded furnace, and thus drying of the moisture in the MSW had to be performed before the incineration process.

### 3.1. Incineration Furnace

To use ash as a partial replacement for cement and aggregates in concrete, preliminary experiments were conducted in a laboratory furnace and a semi-industrial incinerator (detailed in part B), in which MSW destined for landfills was used. The initial incineration temperature of the furnace is 850 °C, and it is increased to ~1000 °C at the end of the process due to the heat produced by the exothermic process.

### 3.2. MSW Incineration in a Laboratory Furnace

**Figure 2:**
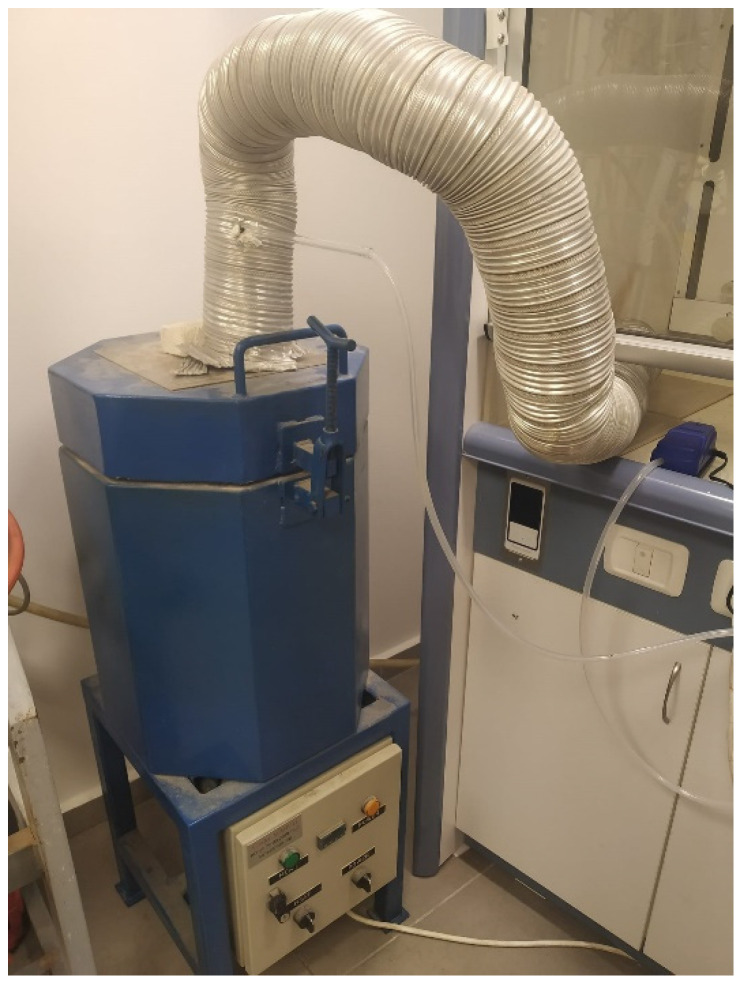
Laboratory furnace.

To conduct initial combustion experiments, a laboratory furnace (Electric Furnaces type 10-12-5 by Adam Mandel company, Be’er Ya’akov, Israel) was upgraded to enable the safe combustion of municipal solid waste. The exhaust gases were transferred through a flexible stainless-steel pipe to the hood. Additionally, the furnace was connected to an air supply unit to ensure a continuous flow of oxygen required for combustion. A photograph of the laboratory furnace during operation is presented in Figure 2.

Before incineration, the MSW was dried in a drying oven at 100 or 130 °C for 2 or 4 h to remove moisture, as shown in Table 1.

**Figure 3:**
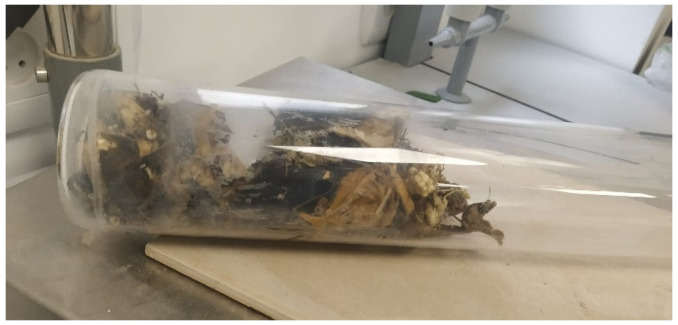
Quartz container with MSW.

The results indicate that the moisture content was in the range of 35–70% with a mean value of 46.1 ± 11.3%. After the drying process, the MSW (100–150 g) was placed in a quartz container (withstanding temperatures of up to 1500 °C, Figure 3), and the quartz container with the MSW was placed in the laboratory furnace for incineration. When the container is inserted into the hot furnace at 850 °C, there is a process of evaporation of gases (seen in the hood at the outlet of the flexible stainless steel pipe) and pyrolysis of the MSW in the quartz container to produce flammable or even explosive gases (such as hydrogen or low molecular weight hydrocarbons, e.g., methane and C2 or C3 gases), resulting in self-ignition and a flame. A series of small explosions occur (these processes occur during the first 4–5 min post insertion of the quartz container, and then the incineration of all the organic content lasts for about 20 min; to ensure complete incineration, the sample was left in the furnace for an additional 2–4 h).

In a typical incineration process experiment, approximately 100 g of MSW was incinerated in the upgraded laboratory furnace at a temperature of 850 °C.

It is planned to add a unique online monitoring of the gas content of the flue gases (e.g., low molecular hydrocarbon gases, carbon monoxide, molecular hydrogen, etc., via mass spectrometry, FTIR spectroscopy, and gas chromatography). However, as the main subject of the present study was the MSW produced, and we have ensured complete oxidation of the organic content of the municipal waste, it is not essential to this study.

### 3.3. Results of the Incineration of the MSW

Table 2 presents the results from MSW combustion experiments conducted in a laboratory furnace, showing that the ash content was, on average, 18% of the original MSW weight. All experiments were performed at the inlet temperature in the range of 815–890 °C. Upon inserting the quartz incineration container into the furnace, the temperature decreases, and it takes several minutes for the temperature to stabilize at the process temperature. Though most of the incineration process occurred in the first 20 min of the procedure (as almost no emission of extra smoke is observed post this period), we have kept the incineration process to 2 or 4 h (Table 2) to achieve complete oxidation of the organic content of the municipal waste.

Table 2 provides additional insights into the behavior of MSW during the combustion process. The wide variation in ash percentages suggests that the heterogeneity of MSW plays a dominant role in determining the final ash yield, more so than incineration time or temperature alone. Although samples incinerated for longer periods (4 h) sometimes produced higher ash percentages, this trend was not consistent across all samples, indicating that differences in waste composition were likely the primary factor. Overall, the average ash content across all samples was linearly correlated with the initial mass of MSW and the ash mass produced, highlighting that the inorganic components of waste primarily determine ash generation. Additionally, while most samples were incinerated at similar temperature conditions, particularly with discharge temperatures approaching 1000 °C, variations in ash yield still occurred, suggesting that even small differences in composition can significantly influence the outcome.

### 3.4. Characterization of the Ash

**Figure 4:**
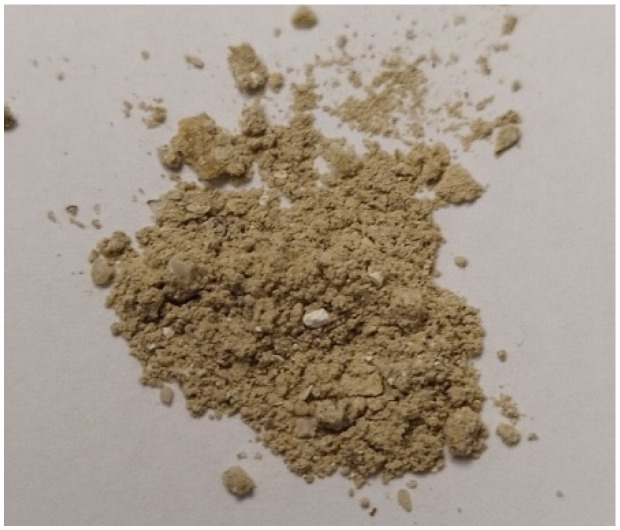
Untreated MSW ash.

The MSW ash consisted of brown particles. A photograph of untreated MSW ash is presented in Figure 4.

Elemental composition analysis was conducted on the ash using an XRF device on a sample of the ash. The results are presented in Table 3.

XRF analysis revealed notable differences in the elemental compositions between samples 1 and 2. Both samples were dominated by calcium, with comparable concentrations (22.7 wt% and 23.5 wt%, respectively). Sample 2 showed higher levels of silicon and magnesium, indicating increased contributions from mineral or glassy components. Elements such as iron and potassium exhibited similar concentrations in both samples, suggesting a consistent source in the waste stream. In contrast, the barium and strontium contents were markedly higher in Sample 2, potentially reflecting the variability in specific waste fractions. Trace-element analysis (PPM) indicated a higher zinc content in Sample 2, whereas manganese and nickel were reported only for Sample 1. Overall, the observed differences suggest heterogeneity in MSW composition.

**Figure 5:**
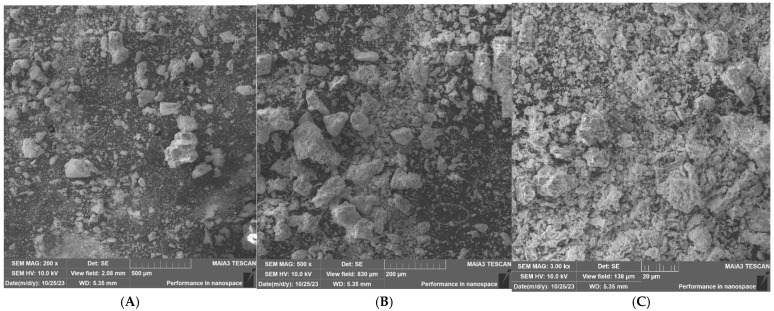
SEM images of MSW ash at various magnifications. (**A**). Magnification of 500 µm, (**B**). Magnification of 200 µm, (**C**). Magnification of 20 µm.

In addition, XRD and SEM analyses were performed and are presented in Table 4 and Figure 5. The XRD table (Table 4) shows the composition of the corresponding compounds found in the analysis.

The XRD results indicated that the main mineral component of the ash (approximately 95%) was calcium minerals composed of anhydrite CaSO_4_, alite 3CaO·SiO_2_, calcite CaCO_3_, and a small amount of quartz SiO_2_ (approximately 5%), which corresponds to the results obtained in XRF (Table 3), in which we observed a large content of Ca. The presence of alite (3CaO·SiO_2_) is particularly noteworthy, as it is a primary constituent of Portland cement, suggesting inherent cementitious properties in the ash.

Figure 5 reveals irregularly shaped particles with a wide distribution, which is characteristic of incinerated waste. Figure 5C (20 µm magnification) shows porous structures and angular fragments, suggesting the potential for mechanical interlocking if used as an aggregate or increased surface area for pozzolanic reactions if finely ground. The absence of identifiable organic residues indicates that the organic content of the MSW was effectively combusted.

### 3.5. Potential Utilization of Treated Municipal Waste Ash in Concrete Blends

A concrete mixture mainly consists of cement, aggregates, and natural sand. Cement is a major and expensive component and a primary source of environmental pollution, with significant carbon dioxide emissions. In addition, fine aggregates and natural sand are essential components of concrete. Owing to their widespread use, these materials have a significant environmental impact. In Israel, there is a significant shortage of fine aggregates. As a by-product, ash has considerable potential to replace natural sand and partially replace cement. Due to its high ash fineness (as shown in Figure 5), ash can partially replace natural sand, thereby increasing the packing density of the concrete and improving the properties of both fresh and hardened concrete. In addition, according to the XRD results (Table 4), the ash, as a combustion product, contains glassy components and therefore can be a good pozzolanic component. It also includes components identical to those of cement and thus can undergo hydration, thereby partially replacing cement. The potential of ash to partially replace sand and cement may improve the properties of fresh concrete, such as increasing density, reducing air content, and enhancing workability, and in addition, improving the properties of hardened concrete, such as increasing its strength due to the pozzolanic and hydration reactions of ash, thereby increasing the CSH content of the concrete. Despite the advantages of using MSW ash, there are several risks that may affect the properties of fresh and hardened concrete. For example, due to the ash’s high porosity, increased water consumption and a corresponding decrease in strength may occur, requiring fine grinding to increase the ash’s reactivity and reduce the risk of ASR. The next article will review the properties of fresh and hardened concrete incorporating ash derived from Municipal Solid Waste (MSW). The performances of the concrete mixtures were tested according to the relevant standards, SI 118 [25], in order to evaluate the possibility of using the concrete mixtures with ash in the concrete industry. However, despite the risks, the potential benefits of using and upgrading the ash to the desired grade represent a significant advantage in the concrete industry.

In other words, using ash as a by-product can be an essential component that reduces concrete costs, improves its technological properties, and mitigates its environmental impact. Furthermore, we have shown in recent publications [26,27,28,29] that thermal or chemical treatments can upgrade the ash quality as a potential substitute for cement and fine aggregates.

## 4. Conclusions

This study confirms the potential of using MSW ash as a partial replacement for cement and aggregates, thereby supporting sustainable construction and circular economy objectives.

The MSW ash has good properties to be utilized as a partial substitute for cement and fine aggregates in concrete blends.

This investigation highlights the environmental benefits of diverting the need to find solutions to MSW ash safe storage in reclamation sites to a good economic solution, thus changing the attitude towards it from an environmental hazard to a useful commodity. Thus, contributing to waste reduction and resource conservation.

In addition, this study successfully established a laboratory-scale incineration process for Israeli MSW, yielding an average ash content of 18%. The resulting ash was characterized by XRF, XRD, and SEM, revealing its primary mineralogical composition to be calcium-based minerals such as anhydrite, alite, and calcite, in addition to quartz. These findings provide a foundational understanding of Israeli MSW ash, paving the way for Part B, which investigates MSW combustion in a semi-industrial incinerator and the performance of MSW ash in concrete blends.

In the second Pt B presentation, the utilization of MSW ash was studied and proved to be a good potential compound for use in concrete blends.

## Figures and Tables

**Table 1:** MSW drying experiments *.

	Weight Before Drying (Grams)	Weight After Drying (Grams)	Temp (°C)	Drying Time (Hours)	Weight Loss (%)	Total Moisture Content of the Sample (%)
1a	120	96	100	2	20	39.4
1b	88	71	100	2	19.4	
2a	90	62	130	2	31.2	60.1
2b	59	42	130	2	28.9	
3a	93	68	100	2	26.9	43.1
3b	68	57	100	2	16.2	
4	100	58	130	2	42	-
5	91	52	100	4	42.9	-
6	277	133	130	4	52	-
7	96	62	130	2	35.5	-
8	213	126	130	2	40.9	-
9	389	116	130	4	70.1	-
10	162	105	130	5	35.2	-

* Samples with the same number and different letter indices: “a” represents the first drying, and “b” represents another drying of the same sample.

**Table 2:** MSW combustion experiments in a laboratory furnace *.

	MSW Weight Before Burning (Grams)	Ash Weight After Burning (Grams)	MSW Inlet Temperature (°C)	Ash Discharge Temperature (°C)	Incineration Time (Hours)	Ash Percentage (%)
1	103	7	890	961	2	6.7
2	153	27	825	964	2	17.6
3	94	16	815	991	4	17
4	110	24	863	1000	4	21.8
5	78	9	877	1000	4	11.5
6	98	32	843	1000	4	32.6
7	114	25	864	1000	4	21.9

* The inlet temperature is the temperature at which the oven stabilizes after sample insertion.

**Table 3:** XRF analysis of untreated MSW ash.

Element	Sample 1	Sample 2
	Weight %	Weight %
Ti	0.37	0.143
Ca	22.7	23.5
Si	4.39	5.41
Cl	1.25	-
Fe	1.13	1.2
P	1.683	-
K	0.323	0.401
Mg	2.2	3.04
S	1.24	-
Al	2.56	2.12
W	0.104	0.104
Ba	0.071	1.58
Sr	0.0585	0.185
Zn	0.00969	0.0115
Ni	0.00052	-
Mn	0.0224	-

**Table 4:** XRD analysis of untreated MSW ash.

Weight Percentage	Non-Treated
Anhydrite—CaSO_4_	37%
Alite—3CaO·SiO_2_	35%
Calcite—CaCO_3_	23%
Quartz—SiO_2_	5%

## Data Availability

The original contributions presented in this study are included in the article. Further inquiries can be directed to the corresponding authors.
